# Evaluation of Structural Stability, Mechanical Properties, and Corrosion Resistance of Magnesia Partially Stabilized Zirconia (Mg-PSZ)

**DOI:** 10.3390/molecules28166054

**Published:** 2023-08-14

**Authors:** Dedek Yusuf, Eneng Maryani, Deby Fajar Mardhian, Atiek Rostika Noviyanti

**Affiliations:** 1 Department of Chemistry, Universitas Padjadjaran, Jl. Raya Bandung Sumedang Km 21, Jatinangor, Sumedang 45361, Jawa Barat, Indonesia; dedek17002@mail.unpad.ac.id; 2 Center for Ceramics, Ministry of Industry of Indonesia, Jl. Ahmad Yani 392, Bandung 40272, Jawa Barat, Indonesia; enengmaryani@kemenperin.go.id; 3 Department of Dental Materials Science and Technology, Faculty of Dentistry, Universitas Padjadjaran, Jl. Raya Bandung Sumedang Km 21, Jatinangor, Sumedang 45361, Jawa Barat, Indonesia; d.fajar.mardhian@unpad.ac.id; 4 Oral Biomaterials Research Center, Faculty of Dentistry, Universitas Padjadjaran, Jl. Sekeloa Selatan I No. 1, Bandung 40132, Jawa Barat, Indonesia

**Keywords:** nano-Mg-PSZ, zirconia, dental implants

## Abstract

Nano Zirconia (ZrO_2_) has been used in dental implants due to having excellent mechanical properties and biocompatibility that match the requirements for the purpose. Zirconia undergoes phase transformation during heating: monoclinic (room temperature to 1170 °C), tetragonal (1170 °C to 2370 °C), and cubic (>2370 °C). Most useful mechanical properties can be obtained when zirconia is in a multiphase form or in partially stabilized zirconia (PSZ), which is achieved by adding small amounts of a metal oxide dopant, such as MgO (magnesia). This study aimed to synthesize nano Mg-PSZ from a local resource found in West Kalimantan, Indonesia, and examine its structural stability, biochemical stability, and mechanical properties. Nano Mg-PSZ was prepared from a zircon local to Indonesia, from West Kalimantan Province, MgSO_4_∙7H_2_O, and polyethylene glycol (PEG)-6000 was used as a template. The obtained *t*-ZrO_2_ after calcination at 800 °C was shown to be stable at room temperature. The highest percentage of the *t*-ZrO_2_ phase was obtained at Zr_0.95_Mg_0.05_O_2_ with a variation of 99.5%. The hardness of Mg-PSZ increased from 554 MPa for ZrO_2_ without MgO doping to 5266 MPa for ZrO_2_ with a doping of 10% MgO. An in vitro biodegradation test showed that the greater the concentration of MgO in doping the ZrO_2_, the greater the degradation resistance of Mg-PSZ in simulated body fluid (SBF) solution.

## 1. Introduction

Zirconia (zirconium dioxide, ZrO_2_), also referred to as “ceramic steel”, has optimal properties for the use of dental implants due to its superior toughness, strength and resistance, excellent wear properties, and biocompatibility [1,2,3]. Amongst the commonly used materials in dentistry, zirconia has the advantage of being compatible for osteoblasts to adhere and proliferate [4]. It is known that the material in dental implant applications must be biocompatible, have antibacterial activity and low toxicity, be stable and resistant to corrosion, and have high performance for survival in the complex mouth environment [5,6,7,8].

Zirconia is known to have three types of crystal: monoclinic (*m*-ZrO_2_), tetragonal (*t*-ZrO_2_), and cubic (*c*-ZrO_2_). The ZrO_2_ phase is unstable and can undergo structural transformations such as *t*-ZrO_2_ to *m*-ZrO_2_ [9]. Metastable tetragonal zirconia is able to transform stress-assisted into the monoclinic state at an ambient temperature if an external load is applied [10]. At room temperature, ZrO_2_ is in the form of a monoclinic phase, whereas to obtain the tetragonal and cubic phases requires a sintering temperature [11]. However, in several syntheses using lower calcination temperatures, tetragonal and cubic phases of ZrO_2_ were obtained [1,12].

Zirconia with tetragonal and cubic structures are generally obtained by adding a stabilizer to the ZrO_2_ lattice structure [13]. Stabilized zirconia generally consist of two or more mixtures of different ZrO_2_ phases and is usually obtained by adding metal oxide dopants such as yttria (Y_2_O_3_) or magnesia (MgO) to the ZrO_2_ lattice [3,14]. Stabilized zirconia is a promising material due to its great physical and chemical properties, and thermal stability [15]. Yttria stabilized zirconia (Y-PSZ) is a zirconia-based dental implant material that is often used. However, Y_2_O_3_ as a ZrO_2_ stabilizer is relatively expensive. Therefore, other metal oxides are needed as zirconia stabilizers. When compared with Y-PSZ, magnesium-stabilized zirconia (Mg-PSZ) shows promising characteristics in several aspects, including good mechanical and thermal properties, good stability in low temperature degradation (LTD), and the same coefficient of thermal expansion as YSZ [16,17,18]. Therefore, Mg-PSZ composite has the potential as a dental implant application material at a relatively low price [1].

As we know, there are three crystallographic phases of zirconia, but it is known that the tetragonal phase (*t*-ZrO_2_) has better mechanical properties and corrosion resistance than the monoclinic and cubic phases [19,20]. In previous studies, *t*-ZrO_2_ was successfully stabilized by adding MgO as a stabilizer and using a low calcination temperature of 800 °C to produce Mg-PSZ. The use of MgO as much as 1, 5, and 10% *w/w* succeeded in stabilizing ZrO_2_ in tetragonal and cubic forms at room temperature [1]. In addition to stabilizing the tetragonal zirconia phase, the addition of 25% MgO concentration increased the hardness (Vickers) of ZrO_2_ from 554 to 6350 MPa and fracture strength from 5.2 to 25 MPa. The increase in the mechanical properties of the sample was caused by the formation of the *t*-ZrO_2_ phase due to the presence of MgO as a stabilizer, which prevents the reverse allotropic transformation of zirconia so as to maintain the *t*-ZrO_2_ phase at room temperature [21]. The lower porosity of the *t*-ZrO_2_ phase compared with the other phases causes the corrosion resistance of *t*-ZrO_2_ to be better than the *m*-ZrO_2_ and *c*-ZrO_2_ phases [19]. The addition of MgO as a stabilizer increased the hardness of ZrO_2_ and gave 50% better wear performance than ZrO_2_ without the addition of MgO [22].

The template method utilizing polyethylene glycol (PEG) is expected to affect the characteristics of ZrO_2_, including changing the morphology of the Mg-PSZ particles. PEG long chains play a role in helping the distribution of metal ions homogeneously and not clumping or settling in the solution so that magnesia-stabilized zirconia is obtained in nano size [14,23]. PEG attaches to the ZrO(OH)_2_ molecule through hydrogen bonds. The hydroxyl group of the ZrO_2_ precursor is covered by a PEG molecule so that aggregation occurring between particles is reduced. During calcination, organic substances are burned as gas, leaving the particles in the nanostructures [14]. This phenomenon reduces the aggregation of synthesized Mg-PSZ particles, so that, in previous studies, Mg-PSZ was obtained with a nano-sized particle diameter of 9–44 nm [1].

In this paper, the mechanical properties and corrosion resistance of Mg-PSZ will be tested. Based on ISO 13356:2015 Third Edition, dental implant materials have a hardness level of 11.8 GPa and the number of monoclinic phases is limited to 20% mass fraction. The Mg-PSZ precursors used in this study were ZrO_2_ derived from local zirconium silicate-based zirconium hydroxide, an MgO stabilizer from a MgSO_4_∙7H_2_O precursor, and polyethylene glycol (PEG) as a template. This research is expected to determine the mechanical properties, corrosion resistance, and antibacterial activity of Mg-PSZ before continuing to direct application as dental implants.

## 2. Results and Discussion

### 2.1. Crystal-Phase Structure Mg-PSZ

The chemical composition of the ZrO(OH)_2_ precursor was first evaluated using the ARL QUANT’X EDXRF Analyzer. Semi-quantitative XRF analysis was carried out to determine the purity of the local zircon and its contents [24]. The results of the XRF analysis shown in Table 1. It showed that ZrO(OH)_2_ contained 79.24 wt% ZrO_2_ and 11.06 wt% MgO, which would be taken into account in the next process to determine each molar ratio of each specimen. The ZrO_2_ was then prepared from ZrO(OH)_2_ after washing with water [1].

**Figure 1:**
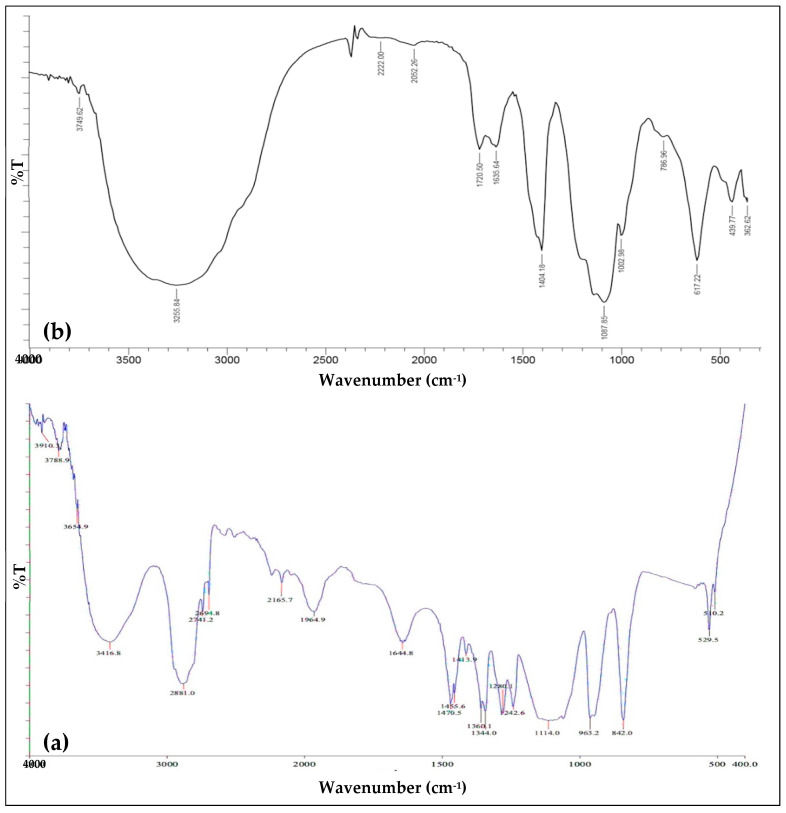
FT-IR spectra of PEG-6000 (**a**) and Zr_0.90_Mg_0.10_O_2_ after drying at 120 °C (**b**) [1].

The mechanism of Mg-PSZ synthesis is the same as in previous studies [1]. The absorption bands in the range of 400–4000 cm^−1^ show several vibrational modes of strain and chemical bonding in the Zr_0.90_Mg_0.10_O_2_ sample and PEG-6000 functional group. Mg-O bonds appear at 617.72 cm^−1^, associated with the stretching vibrations of Mg-O bonds, while the Zr-O bond appears at 439.8 cm^−1^. As shown by the FT-IR analysis in Figure 1, it was observed that the precursor ZrO_2_ reacted with PEG degraded at pH 3 during synthesis, producing Zr-(ethylene glycolate)n and releasing water molecules on heating. PEG hydrogel degradation can occur through hydrolysis due to the presence of strong acids in the form of H2SO4, where the ester bond in the PEG polymer chain will be broken and produce ethylene glycolate as a monomer [25]. The degradation of PEG-6000 at pH 3 was supported by the very low intensity and weak peaks of the CH_2_- strain vibration at 2921.17 cm^−1^ and 2870.738 cm^−1^ compared with the normal PEG which shows a high intensity and strong peaks of the CH_2_ strain vibration around 2890 cm^−1^ [1,2,14,26,27]. Then, heating, which is carried out at a temperature of 120 °C, can cause PEG-6000 to be degraded through a thermal degradation mechanism in which the heat and steam provided will facilitate the PEG’s decomposition [28]. Thermal degradation refers to the breakdown of the molecules of a substance due to overheating, generally related to polymers, in this case PEG-6000 [29].

**Figure 2:**
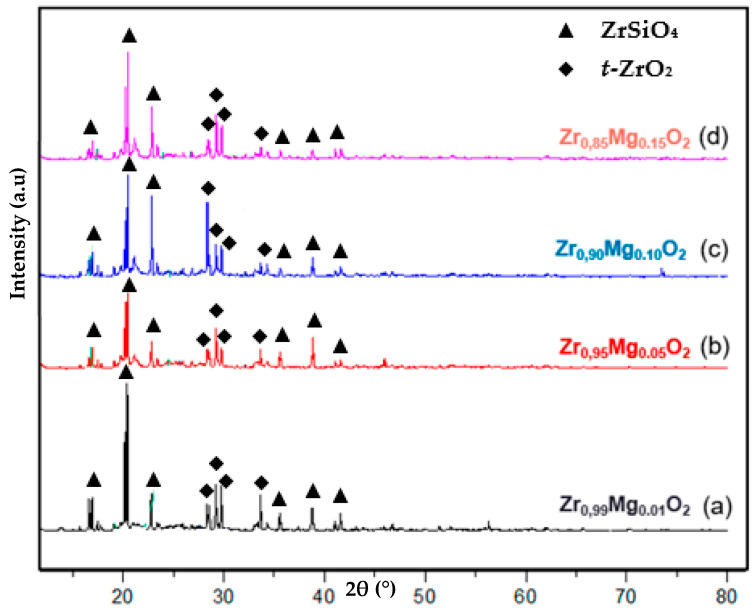
Diffractogram of Mg-PSZ drying 120 °C Zr_0.99_Mg_0.01_O_2_ (a), Zr_0.95_Mg_0.05_O_2_ (b), Zr_0.90_Mg_0.10_O_2_ (c), and Zr_0.85_MgO_0.15_O_2_ (d).

After drying at 120 °C (Figure 2), the XRD analysis results showed a strong peak at 2θ of 20°, indicating the presence of ZrSiO_4_, a commonly found Zr mineral [30]. This specimen was likely derived from the ZrO(OH)_2_, which contained SiO_2_ and led to the formation of ZrSiO_4_. Zirconium silicate is produced from the mineral zircon, which is mined from sand deposits containing several percent zircon and separated by gravity, where it is known as powdered zirconium silicate or as zircon flour [31]. As explained previously, our ZrO_2_ precursor comes from local Indonesian zircon and the purification process carried out in previous studies [1] has not 100% separated the zircon and silicate. Therefore, the presence of the silicates is very likely to occur. Interestingly, we observed the presence of *t*-ZrO_2_, as shown in Figure 2, at the 2θ 30° region. This specimen was confirmed by JCPDS PDF2 no. 791770 and confirmed the previous research that found that the addition of MgO caused the formation of *t*-ZrO_2_. However, our study differs from previous studies on Mg-PSZ composites. We conducted this study using a doping mechanism to obtain *t*-ZrO_2_ and observed changes in the mechanical properties and stability of MgPSZ. The addition of certain stabilizers to the zirconia alloy can help maintain the tetragonal structure at room temperature [32]. The stabilizer used in this research is MgO, which can control the transformation of the stress-inducing phase from *t*-ZrO_2_ to other phases. Based on previous studies regarding the MgPSZ composite, *t*-ZrO_2_ was obtained at 800 °C, but with the doping mechanism in this study at 120 °C, *t*-ZrO_2_ could be formed. However, further testing is required to determine the stability of the *t*-ZrO_2_.

As shown by the XRD analysis in Table 2, the drying at 120 °C only resulted in a low crystallinity of ~50%. The size of the crystallites observed ranged from submicron to micron, with the smallest crystallite of *t*-ZrO_2_ at 164 nm and ZrSiO_4_ at 202 nm, shown in Zr_0.95_Mg_0.05_O_2_, while the largest crystallite was observed in Zr_0.99_Mg_0.01_O_2_, at 771 nm and 4003 nm for *t*-ZrO_2_ and ZrSiO_4_, respectively. This is in line with previous findings that nanoparticle Mg-PSZ was only observed after calcination at a temperature of 600–1000 °C [1]. Magnesium oxide as a stabilizing agent in the preparation of zirconia nano-powders has been demonstrated to have an inhibitory effect on the growth of particle grains and lead to smaller size and more uniform distribution compared with non-stabilized zirconia [33,34].

**Figure 3:**
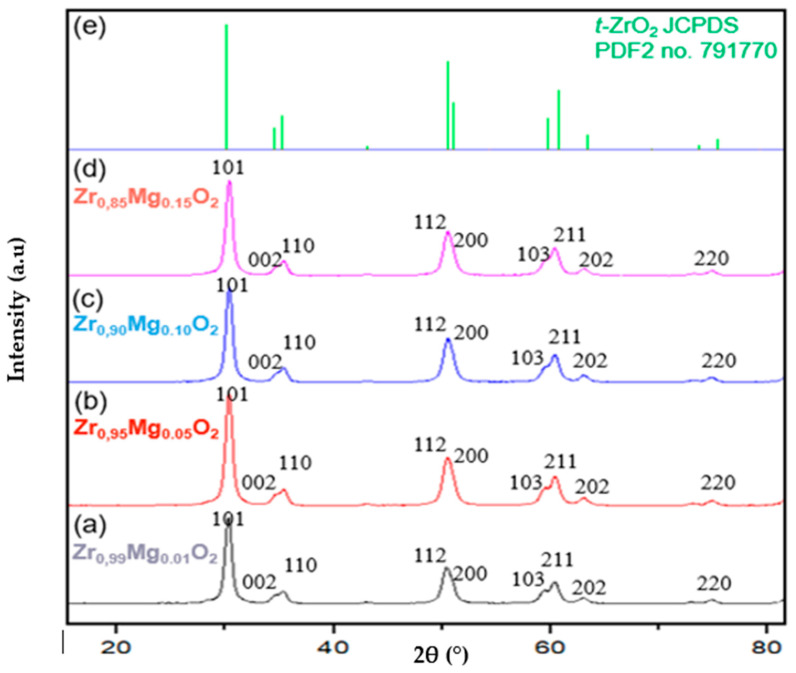
Diffractogram of Zr_0.99_Mg_0.01_O_2_ (a), Zr_0.95_Mg_0.05_O_2_ (b), Zr_0.90_Mg_0.10_O_2_ (c), Zr_0.85_Mg_0.15_O_2_ (d), and *t*-ZrO_2_ JCPDS PDF2 no. 791770 (e).

Next, we observed a structural transformation in ZrO_2_ after calcination, as shown by the XRD analysis in Figure 3. After being calcined at 800 °C, only *t*-ZrO_2_ were observed in all the specimens, as shown in Figure 3. Peaks in all of the Mg-PSZ showed identical principal peaks at 2θ of 30.40°, 34.49°, 35.40°, 50.25°, 50.74°, 59.36°, 60.20°, 62.86, and 74.63°, corresponding to the crystal planes (101), (002), (110), (112), (200), (103), (211), (202), and (220). Those peaks and crystal planes are all associated with *t*-ZrO_2_ (JCPDS PDF2 no. 791770). This is in accordance with a previous study that reported that MgO doping in ZrO_2_ resulted in a *t*-ZrO_2_ structure after calcination at 800 °C [1]. In a former study of Mg-PSZ, a minimum 16% of MgO was required to stabilize ZrO_2_ and form *t*-ZrO_2_, [35]. In another study, MgO at 10% was shown to be sufficient as a stabilizer in obtaining a tetragonal phase [36]. However, we observed that a smaller concentration of MgO at 1% and 5% may also lead to stabilized *t*-ZrO_2_.

The stabilization of the ZrO_2_ structure is caused by cations having a larger radius than Zr^4+^ replacing some of the Zr^4+^ lattice point positions in the ZrO_2_ lattice with doping oxides to become pure ZrO_2_ material [37]. Meanwhile, a substituted solid solution is formed in this ZrO_2_ material through doping, which maintains the stable phase structure of the doped ZrO_2_ material at room temperature, thereby achieving a toughening effect for pure ZrO_2_ materials and leading to the formation of partially stabilized zirconia materials (PSZ) [38,39]. The mechanism of MgO in stabilizing ZrO_2_ can be explained by the difference in charge between the Zr^4+^ ion and the Mg^2+^. The stabilization is caused by a defect in the lattice of a crystal due to doping ions having a lower valence, which leads to oxygen vacancy, as explained in the following equation.

MgO + Zr^4+^ Zr^′^ + ½ O_2_ → Mg^2+^ Zr^′^ + Vo + ZrO_2_ (1)

The reduction of oxygen takes place to balance the positive charge, leading to a neutrally charged Mg-doped ZrO_2_ without free electrons [40]. Oxygen vacancies in the zirconia lattice can reduce the transformation temperature of the transition or metastable phase, and stabilize and increase the concentration of the tetragonal phase in the Zr-ZrO_2_ binary system region [12]. The amount of oxygen vacancies in the ZrO_2_ lattice influences the formation of a different phase of ZrO_2,_ where the tetragonal phase is formed with a low oxygen vacancy, while the cubic phase is formed with a higher oxygen vacancy [18,41].

As shown by the XRD analysis in Table 3, all the specimens of Mg-PSZ calcined at 800 °C have a high crystallinity, with the highest crystallinity of 96.35% being shown in Zr_0.90_Mg_0.10_O_2_ and the lowest at 91.28% shown in Zr_0.99_Mg_0.01_O_2_. The size of the crystallite in all the samples were found to be in a nanometer scale. However, there was an increase in size along with an increase in Mg content, which was likely contributed by Mg.

The overall obtained crystal is *t*-ZrO_2_, as shown in Table 4. The sample Zr_0.95_Mg_0.05_O_2_ has the largest tetragonal phase composition of 99.5%, with a monoclinic phase composition of 0.5% as impurities, while the variation with the lowest tetragonal phase composition is Zr_0.85_Mg_0.15_O_2_ at 96.2% and the monoclinic phase as an impurity is 2.7%. When compared with dental implants with ceramic material yttria-stabilized tetragonal zirconia (Y-TZP) based on ISO 13356:2015 Third Edition, the synthesized partially stabilized magnesia zirconia (Mg-PSZ) meets one of the requirements, which is that the minimum composition of the monoclinic phase is below 20%. Mass fraction has been successfully obtained with very low monoclinic fraction compositions ranging from 0.5 to 2.7% for all synthesized Mg-PSZ.

### 2.2. Mechanical Properties of Mg-PSZ

Hardness is one of the most important parameters for comparing dental implant material properties, as it is used to find the suitability of the clinical use of biomaterials [42]. A study explained that the addition of 10% MgO concentration increased the hardness of Vickers ZrO_2_ from 554 to 6350 MPa [20]. The increased mechanical properties in *t*-ZrO_2_ was due to the presence of MgO, which prevents the reverse allotropic transformation of zirconia. In general, an increase in hardness that requires a decrease in porosity is known as the Duckworth–Knudsen model [43]. The results of the hardness test are shown in Table 5.

**Figure 4:**
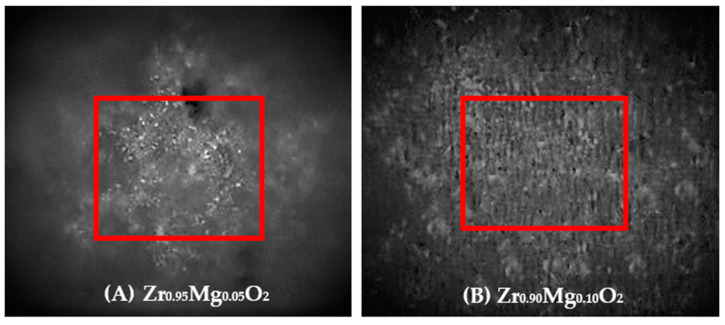
Magnification of 100× microvickers, Zr_0.95_Mg_0.05_O_2_ (**A**) and Zr_0.90_Mg_0.10_ O_2_ (**B**).

The Vickers hardness test showed that doping MgO in ZrO_2_ successfully increased the hardness of the ZrO_2_, as shown in Table 5. Zr_0.90_Mg_0.10_O_2_ has shown the highest Vickers hardness with a value of 5266 MPa. This obtained result is almost in accordance with a previous study that reported an increase in hardness to 6350 MPa after an addition of 10% MgO in ZrO_2_ [20]. However, we did not observe a consistent trend in the increase in hardness. This is most likely due to the different surface roughness of each specimen [44]. As shown in Figure 4, the Zr_0.95_Mg_0.05_O_2_ sample showed a less flat surface, while in contrast, Zr_0.90_Mg_0.10_O_2_ showed a flat surface. This led to different pressures in the indenter when the test was carried out. When a rough surface is stressed, the resulting pressure triangle will produce a very large distance, which will cause the calculation of the Vickers hardness value to be small. Meanwhile, lower roughness leads to a smaller triangle size and the Vickers hardness value being large.

### 2.3. Stability of Mg-PSZ

**Figure 5:**
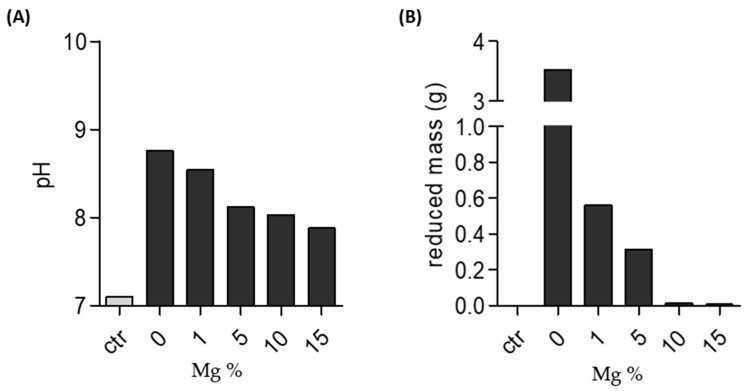
Stability of Zirconia and Mg-PSZ. (**A**) pH of solution after zirconia or Mg-PSZ soaked for 3 days in SBF. (**B**) Loss of weight after zirconia or Mg-PSZ soaked for 3 days in SBF. SBF solution without zirconia or Mg-PSZ was used as control.

The stability test of Mg-PSZ was carried out by a simple in vitro biodegradation test. Each specimen was immersed in SBF (Simulated Body Fluid) solution for 3 days. As shown in Figure 5, the pH of the SBF solution was changed after the ZrO_2_ and Mg-PSZ samples were soaked for 3 days at 37 °C.

In general, the dissolution reaction of ZrO_2_ in an aqueous medium follows the equation [45]:

ZrO_2_(c) + (4 − n) H^+^ → Zr(OH)_n_^4−n^ + (2 − n)H_2_O (2)

Soaking the sample for 3 days in SBF solution at 37 °C showed a change in pH, as shown in Figure 5A. After soaking for 3 days, a significant change in pH was found in the ZrO_2_, which was at 8.76, and gradually lower changes in pH were observed in samples with increasing MgO. Additionally, this changes in pH corresponded with mass loss, as shown in Figure 5B. Thus, the changes in pH of the SBF solution in the sample is due to the release of Zr^4+^ ions from the ZrO_2_.

The immersion of the ZrO_2_ and the variation of the Mg-PSZ carried out for 3 days at 37 °C showed the largest mass change for the ZrO_2_ without MgO doping with a mass loss of 3.2545 g. Testing the variation of the Mg-PSZ sample in Figure 5B shows that the degradation of the sample that occurred is strongly influenced by the concentration of the MgO used. Sample variation 4, with the addition of 15% of MgO, showed the best resistance of the material to SBF with a lost weight of 0.0069 g. These data show a correlation between changes in SBF pH and the amount of ZrO_2_ sample dissolved in the SBF solution.

Based on the variation of MgO concentrations, we concluded that the greater the concentration of MgO in doping the ZrO_2_, the greater the degradation resistance of the SBF solution. However, another thing to note is that although the addition of MgO showed a significant effect on ZrO_2_ resistance, further tests (in vivo tests) were needed to determine the time of osteoblast formation in the bone and the effect of pH on osteoblast cells. This is because changes in pH in the osteoblast cell environment can provide an inflammatory response so that the formation of osteoblast cells becomes slow [46].

## 3. Materials and Methods

Materials used were (NH_4_)HCO_3_ (PT. Brataco, Jakarta, Indonesia), H_2_SO_4_ 95–97% (Merck KGaA, Darmstadt, Germany), Carboxyl Methyl Cellulose (PT. Brataco, Jakarta, Indonesia), MgSO_4_∙7H_2_O (Merck KGaA, Darmstadt, Germany), PEG-6000 (PT. Brataco, Jakarta, Indonesia), Simulated Body Fluid (SBF) (MaxLab, Jakarta, Indonesia), and zirconium oxyhydroxide (ZrO(OH)_2_) prepared from local zirconium silicates from the province of West Kalimantan, Indonesia. First, the Mg-PSZ was synthesized from ZrO(OH)_2_, MgSO_4_·7H_2_O as dopant, and PEG-6000 for template. The obtained structure of Mg-PSZ was then analyzed using XRD. Next, physical properties were analyzed by means of hardness and stability in biological environment.

### 3.1. Preparation of Crystals

Prior to the synthesis, zirconium oxyhydroxide was analyzed using XRF to determine the ZrO_2_ content. Next, 60 g of ZrO(OH)_2_ was dissolved in 25 mL of 17.74 M H_2_SO_4_. The pH was adjusted to 3.0 by adding appropriate amount of 1 M (NH_4_)HCO_3_. Subsequently, MgSO_4_∙7H_2_O was added in a molar ratio of 0.01, 0.05, 0.10, or 0.15 compared with Zr. Afterwards, 10% PEG-6000 in water was added to the mixture of Mg:Zr at a volume ratio of 1:15 (PEG-6000:Mg-Zr). Mixture was continuously stirred until homogeneous for 1 h, then heated at 120 °C for 3 h and rested overnight at RT. The next day, gel was filtered and followed by drying at 100 °C for 2 days to obtain dry solid material. Finally, solid material was rinsed with hot deionized water to remove impurities and then dried again at 100 °C for 1 day. After drying, powder was collected for FTIR, XRD, and further processing. Powder was then calcined at 800 °C and further characterized using XRD and TEM [1,2].

### 3.2. Mechanical Properties

To evaluate the hardness of Mg-PSZ, material was made into a 2 cm × 2 cm × 1 cm block for microvickers test. The load was set at 200 g.f for 10 s. Vickers hardness was then calculated using the following formula [47]:

(3) $$HV=\frac{2P\sin\left(\frac{\alpha }{2}\right)}{d^{2}}$$

HV = Vickers Hardness, P = Load (kg.f), α = opposite angle of the indenter, and d = the average indentation diagonal.

### 3.3. Stability

To examine the stability in a biological setting, Mg-PSZ was soaked in a simulated body fluid, followed by measurement of pH in the solution and changes in specimen weight, according to previous study [48]. Briefly, prepared Mg-PSZ block was soaked in a simulated body fluid which has very similar ionic composition to human blood plasma. The ion contents are shown in Table 6. After soaked for 3 days at 37 °C, each Mg-PSZ was weighted to measure changes compared with initial weight before soaking. Furthermore, pH of SBF before and after the experiment was measured.

## 4. Conclusions

The introduction of MgO led to the creation of *t*-ZrO_2_. A heating process at 800 °C enhanced the structural crystallinity and further stabilized ZrO_2_. While the utmost *t*-ZrO_2_ composition (99.5%) was attained with 5% MgO (Zr_0.95_Mg_0.05_O_2_), the ZrO_2_ sample containing 10% MgO (Zr_0.95_Mg_0.05_O_2_) exhibited the highest level of crystalline quality at 96.35%. Moreover, ZrO_2_ containing 10% MgO exhibited the highest Vickers hardness at 5266 MPa. Conversely, elevated concentrations of MgO resulted in larger crystal sizes and improved resistance in biological environments.

## Figures and Tables

**Table 1:** XRF analysis of ZrO(OH)_2_ precursors.

Compound	Weight %
ZrO_2_	79.2400
MgO	11.0600
SiO_2_	7.7600
HfO_2_	0.5780
MoO_3_	0.5700
Y_2_O_3_	0.4230
Cs_2_O	0.1660
Fe_2_O_3_	0.0344
CdO	0.0339
CuO	0.0331
K_2_O	0.0290
Ag_2_O	0.0205
SrO	0.0201

**Table 2:** Crystallinity and crystallite size of Mg-PSZ dried at 120 °C.

Samples	Crystallinity (%)	Crystal Size ZrSiO_4_ (nm)	Crystal Size *t*-ZrO_2_ (nm)
Zr_0.99_Mg_0.01_O_2_	53.56	4002	771
Zr_0.95_Mg_0.05_O_2_	51.70	202	164
Zr_0.90_Mg_0.10_O_2_	52.41	425	193
Zr_0.85_Mg_0.15_O_2_	50.87	2434	225

**Table 3:** Crystallinity and crystallite size of calcined Mg-PSZ 800 °C.

Sample	Crystallinity (%)	Crystal Size *t*-ZrO_2_ (nm)
Zr_0.99_Mg_0.01_O_2_	91.28	78
Zr_0.95_Mg_0.05_O_2_	95.32	81
Zr_0.90_Mg_0.10_O_2_	96.35	97
Zr_0.85_Mg_0.15_O_2_	95.85	112

**Table 4:** Mg-PSZ phase percentage.

Samples Mg-PSZ	Monoclinic (%)	Tetragonal (%)
Zr_0.99_Mg_0.01_O_2_	2.1	97.9
Zr_0.95_Mg_0.05_O_2_	0.5	99.5
Zr_0.90_Mg_0.10_O_2_	2.4	97.6
Zr_0.85_Mg_0.15_O_2_	2.7	96.2

**Table 5:** Vickers hardness of ZrO_2_ and Mg-PSZ.

Samples	Hardness (HV)	Hardness (MPa)
ZrO_2_ [21]	59.5	554
Zr_0.99_Mg_0.01_O_2_	407	3991
Zr_0.95_Mg_0.05_O_2_	98.9	969.9
Zr_0.90_Mg_0.10_O_2_	537	5266
Zr_0.85_Mg_0.15_O_2_	125	1226

**Table 6:** Comparison of ions in SBF and blood plasma in the body [49].

	Ion Concentration (mM)						
	Na^+^	K^+^	Mg^2+^	Ca^2+^	Cl^−^	HCO_3_^−^	HPO_4_^−^
Plasma	142.0	5.0	1.5	2.5	103.0	27.0	1.0
SBF	142.0	5.0	1.5	2.5	148.8	4.2	1.0

## Data Availability

Not applicable.
